# Mapping quantitative trait loci for heat tolerance of reproductive traits in tomato (*Solanum lycopersicum*)

**DOI:** 10.1007/s11032-017-0664-2

**Published:** 2017-04-18

**Authors:** Jiemeng Xu, Nicky Driedonks, Marc J. M. Rutten, Wim H. Vriezen, Gert-Jan de Boer, Ivo Rieu

**Affiliations:** 1 Department of Molecular Plant Physiology, Radboud University, 6525 AJ Nijmegen, The Netherlands; 2 Bayer Vegetable Seeds, 6083 AB Nunhem, The Netherlands; 3 ENZA Zaden Research and Development B.V, 1602 DB Enkhuizen, The Netherlands

**Keywords:** QTL mapping, Reproductive heat tolerance, Pollen viability, Tomato

## Abstract

Global warming has become a worldwide concern due to its adverse effects on agricultural output. In particular, long-term mildly high temperatures interfere with sexual reproduction and thus fruit and seed set. To uncover the genetic basis of observed variation in tolerance against heat, a bi-parental F_2_ mapping population from two contrasting cultivars, i.e. Nagcarlang and NCHS-1, was generated and phenotyped under continuous mild heat conditions for a number of traits underlying reproductive success, i.e. pollen viability, pollen number, style length, anther length, style protrusion, female fertility and flowering characteristics, i.e. inflorescence number and flowers per inflorescence. Quantitative trait loci (QTLs) were identified for most of these traits, including a single, highly significant one for pollen viability, which accounted for 36% of phenotypic variation in the population and modified pollen viability under high temperature with around 20%. QTLs for some traits colocalised, indicating trait dependency or pleiotropic-effect loci. We conclude that a limited set of major genes determines differences in performance of reproductive traits under continuous mild heat in tomato. The results contribute to our fundamental understanding of pollen thermotolerance and may support development of more heat-tolerant tomato varieties.

**Electronic supplementary material:**

The online version of this article (doi:10.1007/s11032-017-0664-2) contains supplementary material, which is available to authorized users.

## Introduction

High temperature is one of the major abiotic stress factors affecting plants, having adverse effects on both growth and reproduction. In an agricultural context, this leads to negative effects on yields (Jha et al. 2014; Driedonks et al. 2016). Temperature projections for the coming century show a continuing increase in global surface temperature and suggest more frequent and more severe heat waves, meaning that crops are even more likely to be exposed to high temperature during their growth period (Pachauri et al. 2014). For instance, the maize cultivation area that suffers from stress-inducing levels of heat has been predicted to increase from 15% in 2000 to 44% in 2050 (Gourdji et al. 2013). Therefore, a better understanding of plant heat tolerance mechanisms and description of existing heat tolerance traits is urgently needed.

A number of basic processes, such as protein folding, maintenance of membrane stability, photosynthesis and assimilate metabolism, are shown to be affected by heat (Bokszczanin et al. 2013). When considering long-term mildly high temperatures, representative of heat waves, life cycle stages clearly differ from each other regarding their sensitivity, with reproductive processes found to be more vulnerable than vegetative ones (Hall 1992). This vulnerability applies especially to the meiotic to early microspore stages during pollen development, and early embryo development at 1–3 days after fertilisation (Hedhly et al. 2009; Giorno et al. 2013; Bac-Molenaar et al. 2015; Müller and Rieu 2016). There seems to be considerable natural variation for heat tolerance in reproduction within plant species (Shpiler and Blum 1986; Patel and Hall 1990; Redona et al. 2009; Bac-Molenaar et al. 2015) and QTLs for a number of underlying traits have been identified (Esten Mason et al. 2011; Ye et al. 2012; Bac-Molenaar et al. 2015). The underlying causal genes and their physiological effects have not yet been reported.

As an important horticultural crop grown for fruit production in subtropical climates, tomato has been extensively studied for heat tolerance at reproductive phase. Similar to other plant species, the reproduction is particularly sensitive to continuous mild heat (CMH; Kinet and Peet 1997). Under these conditions, male fertility and the position of the stigma relative to the anther cone seem to be major factors limiting fruit and seed set (Dane et al. 1991; Levy et al. 1978). A number of studies have assessed tomato cultivars for fruit set under CMH condition and identified relatively well-performing genotypes (Levy et al. 1978; Dane et al. 1991; Sato et al. 2000; Sato et al. 2004; Bhattarai et al. 2016). The largest, multi-year characterisations by the Asian Vegetable Research and Development Center (AVRDC) identified 39 tolerant lines, some of which have already been utilised in tomato breeding programs (Opeña et al. 1992; Scott et al. 1995; Gardner 2000). However, to be able to efficiently use variation in heat tolerance for fundamental and applied aims, it is necessary to have knowledge on the genetic basis of the trait. Tomato QTLs associated with reproduction under heat were reported in two studies (Grilli et al. 2007; Lin et al. 2010), but identified markers and linkage groups were not associated with chromosomes, hampering wider use of the findings.

The objective of this study was to dissect the genetic architecture underlying tolerance of key reproductive traits to CMH. Tolerance regarding pollen viability was identified in tomato cultivar Nagcarlang, while tolerance with respect to total pollen number and stigma protusion was identified in cultivar NCHS-1. Here, in a forward genetic approach, an intraspecific F_2_ population derived from these two phenotypically contrasting parents was used for QTL mapping.

## Material and methods

### Plant material

Tomato (*Solanum lycopersicum*) cultivars Nagcarlang (LA2661) and NCHS-1 (LA3847) were obtained from TGRC and crossed by using Nagcarlang as the mother plant. F_2_ seeds were collected from a single F_1_ individual, and 180 F_2_ individuals were phenotyped for QTL analysis.

### Plant husbandry and heat stress phenotyping

Seeds of the two parental cultivars and the F_2_ population were sown in standard commercial potting soil (Lentse Potgrond number 4, Horticoop, Katwijk, The Netherlands) with vermiculite scattered on top to cover the seeds. Ten days after sowing, seedlings were transplanted into separate pots with commercial potting soil and at 20 days after sowing, seedling were transferred further to 12-l pots filled with the same soil supplemented with slow-release fertiliser (4 g L^−1^ Osmocote Exact Standard 3–4 M, Everris International B.V., Geldermalsen, The Netherlands). Plants were grown under standard greenhouse conditions with a 16-h light period (supplemented with artificial light from 600 W sodium lamps if natural light intensity fell below 250 μmol m^−2^ s^−1^) and a temperature of around 25 °C in the day (minimum set to 20 °C) and 19 °C in the night (minimum set to 17 °C).

When the first inflorescences were detectable by eye, all inflorescences were removed and plants were transferred to climate chambers with CMH condition (31 °C day, 25 °C night; 14 h light, [∼250 μmol m^−2^ s^−1^ at plant height, Philips fluorescent lamps], 10 h dark; 70–80% RH). The following phenotypical data were collected: inflorescence number (IN; i.e. number of inflorescences on top 5 branches present 3 weeks after start of CMH), flowers per inflorescence (FPI; i.e. average number of flowers on 3 randomly chosen inflorescences), anther length (AL; i.e. of 6 to 10 newly opened flowers), style length (SL; i.e. of 6 to 10 newly opened flowers), style protrusion (SP = SL–AL; i.e. of 6 to 10 newly opened flowers), pollen viability (PV; i.e. in-vitro pollen germination percentage of 6 to 10 newly opened flowers), pollen number (PN; i.e. of 6 to 10 newly opened flowers) and female fertility (FF). To assess PV, anthers from newly opened flowers were cut into four pieces and pollen was incubated under wet condition for 30 min. Hydrated pollen were released by vortexing in 0.5 mL artificial germination medium (25% (*w*/*v*) PEG 4000, 5% (*w*/*v*) sucrose, 1 mM KNO_3_, 1 mM Ca(NO_3_)_2_·4H_2_O, 1.6 mM H_3_BO_3_, 0.8 mM MgSO_4_·7H_2_O) and further incubated at room temperature for 1.5 h under constant rotation. Subsequently, the pollen suspension was loaded on a haemocytometer and pollen with tubes longer than the diameter was rated as germinated; about 100 pollens were evaluated for calculating germination rate. The number of pollens in 25 squares (0.1 μL) of the haemocytometer were counted, and values were expressed as PN. After phenotype evaluation under CMH, plants were moved back to normal greenhouse conditions and four open flowers were immediately pollinated with NCHS-1 pollen from control condition. Seed number of fruits from hand pollination were counted to evaluate FF.

To confirm QTL qPV11, 31 F_3_ progenitor plants from single F_2_ plant were genotyped at the closest marker and phenotyped for PV under CMH using impedance flow cytometry with AMPHA Z30 (Amphasys AG, Lucerne, Switzerland). Per plant, pollen was analysed at four different days with three fresh flowers pooled as one sample per day.

### SNP genotyping

Single-nucleotide polymorphisms (SNPs) between Nagcarlang and NCHS-1 were identified using the SolCAP SNP array, according to Sim et al. (2012). Ninety-six SNPs (Supplementary Table 1), relatively evenly distributed over the tomato genome, were used for genotyping by KASP assays (LGC, Teddington, UK; Supplemental Table 1) according to the manufacturer’s instructions.

### Statistical and QTL analysis

Without explicit mention, all statistical analyses were done with R (version 3.2.0, R Core Team 2015). Phenotypic differences between the two parental cultivars were compared by Welch (unequal variance assumption) two-sample *t*-test with *t.test* function. In addition, the descriptive statistics of F_2_ population were calculated and trait frequency distributions were plotted. Pearson correlations between mother plants (F_2_ plants) and cuttings, and among phenotypic traits in the F_2_ population were calculated and visualised by using the Hmisc package (Frank and Harrell 2015). PV data from the F_3_ progenitor plants were analysed by ANOVA and Tukey post hoc test.

Phenotypic and genotypic data were integrated for QTL mapping with the R/qtl package (Broman et al. 2003). Marker diagnostics was performed, and 12 low-quality markers were discarded. Furthermore, one marker assay showing strong segregation distortion (*P* < 0.0001) and two marker assays showing high correlation with segments of different chromosomes were excluded. A genetic map was constructed by using the *est.map* function based on 81 informative markers. Standard interval mapping (SIM) and composite interval mapping (CIM) were applied for QTL detection. For SIM, the whole genome was scanned at steps of 1 cM by by using the *scanone* function with batch and climate chamber added as covariates. CIM was performed by using the *cim* function at 1 cM step, and three markers were selected as cofactors by forward selection. Expectation-maximisation was chosen as the solution-generating algorithm; other settings were left at their default values. Significance thresholds were generated for each trait by using the permutation test (α = 0.05, *n* = 1000; Churchill and Doerge, 1994). Both for mapping construction and QTL analysis, the Kosambi map function was used for the conversion of recombination frequency to genetic distance (Kosambi 1944). Since highly similar results were obtained from SIM and CIM, only CIM results were further analysed by using the *fitqtl* function to obtain the effects and interactions of QTLs.

## Results

### Phenotypic variation under CMH condition

As tomato seed and fruit set under high temperature are complex traits, which reduces the power of QTL mapping, we analysed various subtraits known to contribute to reproductive success under high temperature, i.e. PV, PN, AL, SL, SP from the anther cone and FF. The tomato cultivars Nagcarlang and NCHS-1 were found to contrast for a number of these traits when grown under CMH (Table 1). A bigger portion of the pollen from Nagcarlang was viable, whereas NCHS-1 produced a larger number of pollen, had a shorter style and less style protrusion. Moreover, Nagcarlang flowered significantly earlier, produced more inflorescences and formed more flowers per inflorescence than NCHS-1 under CMH.

**Table 1:** Phenotype of the two parental lines and F_2_ population under continuous mild heat condition

	Nagcarlang		NCHS-1		F_2_ population						
Trait	*n*	Mean	*n*	Mean^a^	*n*	Mean	SD	Minimum	Maximum	Kurtosis	Skewness
PV (%)	20	35.6	21	21.1**	180	25.7	11.4	2.7	61.8	0.71	0.51
PN (× 5000)	20	11.9	21	17.4**	180	13.1	4.3	4.2	28.1	0.73	0.87
FF	14	113.5	9	81.8*	122	119.7	28.3	22.0	185.5	0.51	−0.23
SP(mm)	20	1.2	21	0.2**	180	1.0	0.7	−0.7	3.3	−0.08	0.32
SL (mm)	20	7.9	21	6.7**	180	7.9	0.8	5.6	10.1	0.06	−0.02
AL (mm)	20	6.7	21	6.5	180	6.9	0.5	5.4	8.1	−0.12	0.05
IN	20	24.8	18	12.2**	180	16.0	3.5	7.0	24.0	−0.64	0.03
FPI	20	5.8	19	4.1**	180	5.2	0.9	2.7	7.7	−0.14	0.01

*PV* pollen viability, *PN* pollen number, *FF* female fertility, *SP* style protrusion, *SL* style length, *AL* anther length, *IN* inflorescence number, *FPI* flowers per inflorescence

*Significantly different from Nagcarlang, *P* ≤ 0.01; **P ≤ 0.001

^a^Differences between the means of Nagcarlang and NCHS-1 were compared by Welch’s *t*-test

In the F_2_ population, phenotypic data for all traits scattered over wide continuous ranges, indicating quantitative inheritance (Supplementary Fig. 1). FPI, AL and SL followed normal distributions as their kurtosis and skewness values were close to 0. Distributions of the other traits skewed somewhat to the right except for FF (Table 1, Supplementary Fig. 1). Transgressive segregation was observed for AL and FF since the F_2_ population mean for these two tratis was outside the ranges defined by the two parental population means (Table 1).

Reliability of the phenotyping assay was verified by correlating phenotypic data on PV, PN, SL, AL and SP between 35 F_2_ individuals and clones thereof. For all traits, strong and positive correlations were found, in particular for PV and SP (Supplementary Fig. 2). This indicates that in the used setup, these traits showed moderate to high heritability.

### Trait correlations in the F_2_ population

**Fig. 1:**
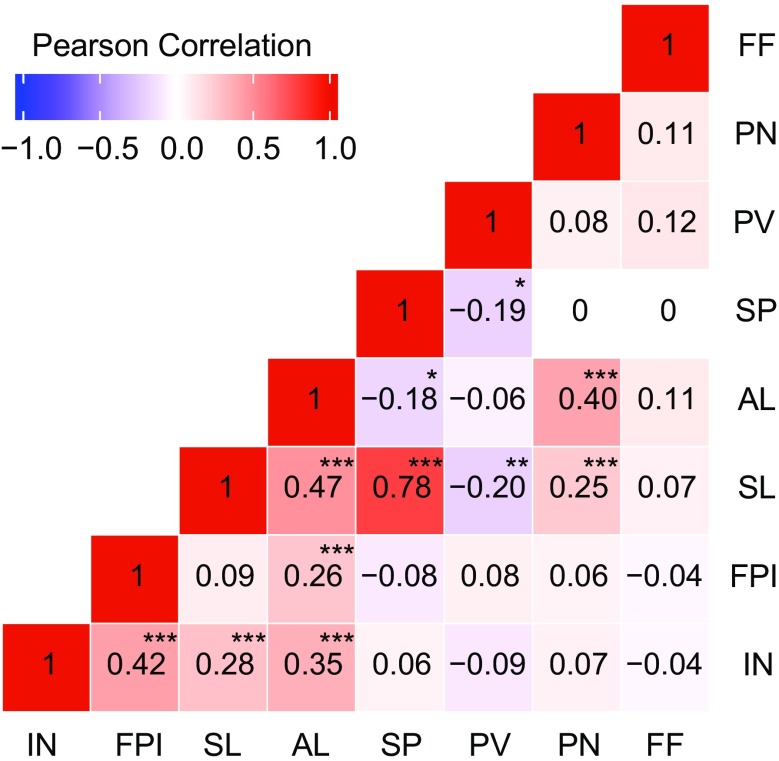
Pearson correlations among phenotypic traits in the Nagcarlang × NCHS-1 F_2_ population. Significance level: *P ≤ 0.05; **P ≤ 0.01; ***P ≤ 0.001. Trait abbreviations: IN, inflorescence number; FPI, flowers per inflorescence; SL, style length; AL, anther length; SP, style protrusion; PV, pollen viability; PN, pollen number; FF, female fertility

To assess associations among traits, Pearson correlation analysis was applied (Fig. 1). For the size of floral organs, positive correlations were found between SP and SL, and between SL and AL. AL was also positively correlated with PN (Fig. 1). Also, a number of relatively weak associations were observed, such as the negative correlations between PV and SL, SP. Female fertility was not correlated to any other trait and therefore might be considered as an independent trait. In addition, positive correlation was found between IN and FPI.

### QTL analysis and their interaction

**Fig. 2:**
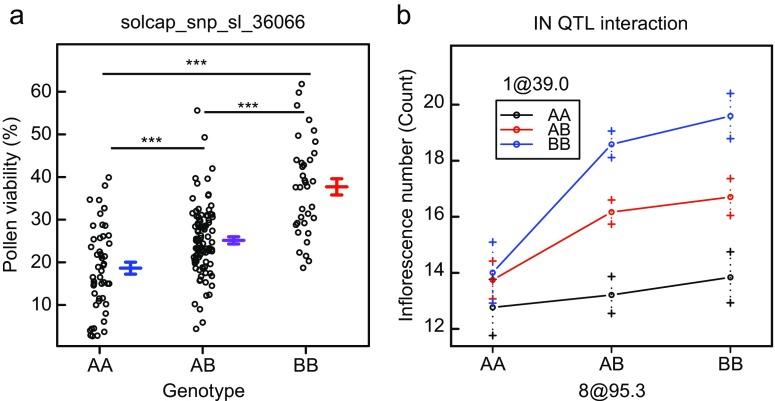
Genotype effects on pollen viability and inflorescence number QTL interaction effect. **a** Comparing PV by genotypes at the closet marker to qPV11. The A allele originates from NCHS-1 and the B from Nagcarlang. **b** Interaction of two QTLs for inflorescence number (IN). If one of the QTLs is homozygous to NCHS-1, the effect of the other one is small

A single, highly significant QTL associated with pollen viability (qPV11) was detected on chromosome 11 and explained 36.3% of phenotypic variation (Table 2, Supplementary Fig. 3). The additive and dominance effects of qPV11 were 9.1 and −3.3, respectively. The allele contributing positively to pollen viability originated from Nagcarlang. PV of the three qPV11 genotypes, according to the closest marker, differed significantly from each other (Fig. 2a). Likewise, a QTL for PN was identified on chromosome 7 (qPN7) and explained 18.6% of the phenotypic variation of the trait (Table 2, Supplementary Fig. 3). The positive effect was contributed by the NCHS-1 allele. The additive and dominance effects were −2.4 and 0.3, respectively.

**Table 2:** Overview of mapped QTLs

Trait	QTL	Composite interval mapping (CIM)					
		Position^a^	Closest SNP	LOD	a	d	*R* ^2^ (%)
PV	qPV11	11@19.4	solcap_snp_sl_36066	17.6	9.1	−3.3	36.3
PN	qPN7	7@134.7	solcap_snp_sl_12139	8.0	−2.4	0.3	18.6
SP	qSP1	1@16	solcap_snp_sl_8704	8.5	0.3	0.1	19.5
	qSP3	3@80.4	solcap_snp_sl_7942	12.9	0.4	0.2	28.0
AL	qAL1	1@70	solcap_snp_sl_42213	6.6	0.3	0.0	15.5
	qAL2	2@80.8	solcap_snp_sl_36287	4.8	−0.2	0.0	11.6
	qAL7	7@134.7	solcap_snp_sl_12139	11.3	−0.3	0.1	25.2
SL	qSL1	1@16	solcap_snp_sl_8704	10.1	0.4	0.0	22.7
	qSL2	2@80.8	solcap_snp_sl_36287	4.3	−0.3	0.0	10.5
	qSL3	3@75.8	solcap_snp_sl_7942	6.7	0.3	0.3	15.8
FPI	qFPI1	1@40	solcap_snp_sl_13762	19.1	0.7	0.0	38.7
IN	qIN1	1@39	solcap_snp_sl_13762	9.7	2.1	0.2	21.9
	qIN8	8@95.3	solcap_snp_sl_15446	5.6	1.7	0.8	13.4

*PV* pollen viability, *PN* pollen number, *SP* style protrusion, *AL* anther length, *SL* style length, *FPI* flowers per inflorescence, *IN* inflorescence number, *a* additive effect (positive value indicates a positive effect from Nagcarlang, negative value indicates a positive effect from NCHS-1), *d* dominance effect

^a^Postition is presented as chromosome@genetic position

QTLs for SL, AL, SP, IN and FPI were also identified (Table 2, Supplementary Fig. 3). The two QTLs for SP, on chromosomes 1 (qSP1) and 3 (qSP3), colocalised with two QTLs for SL (qSL1, qSL3), while a third QTL for SL (qSL2) colocalised with a QTL for AL (qAL2). Of the two additional QTLs for AL, the stronger one (qAL7) colocalised with qPN7 for PN. Two QTLs were found for IN on chromosomes 1 and 8. Among the traits with multiple QTLs, only those for IN interacted: if qIN1 was homozygous for the NCHS-1 allele, the effect of qIN8 was relatively small, whereas it became larger with qIN1 having one or two Nagcarlang alleles (Fig. 2b).

### Validation of the QTL for pollen viability heat tolerance

Thirty-one F_3_ plants were generated from a single F_2_ plant that was heterozygous for qPV11. Plants homozygous for the Nagcarlang allele of qPV11 produced pollen with significant higher viability than those homozygous for the NCHS-1 allele and heterozygous plants (Table 3). No difference was found between the latter two genotypes.

**Table 3:** QTL validation for pollen viabiliy under CMH

Genotype^a^	Number	Pollen viability^b^
AA	16	47.7 ± 2.6a
AB	9	40.9 ± 2a
BB	6	71 ± 7.6b

^a^F_3_ plants were grouped into three genotypes according to the closet marker to qPV11. “A,” allele from NCHS-1; “B,” allele from Nagcarlang

^b^Pollen viability of the three genotypes was shown as “mean ± SE” and labelled with letters for significant difference (*P* ≤ 0.001)

## Discussion

### Pollen viability and number

Pollination with a sufficient number of viable pollen is required for effective fertilisation in flowering plants. The number of viable pollen depends on the total number of pollen that are available at flower anthesis and the portion of them that are able to germinate and form a pollen tube. Both these factors were significantly decreased when tomato plants were grown under a long-term mild heat conditions. This is in accordance with previous reports, a number of which also showed a positive correlation between pollen performance and fruit set under a similar type of high-temperature regime (Akhtar et al. 2012; Dane et al. 1991; Firon et al. 2006; Levy et al. 1978; Pressman et al. 2002; Sato et al. 2000; Sato et al. 2006). In the present study, the tomato accession Nagcarlang was found to have relatively high pollen thermotolerance, in agreement with findings of Dane et al. (1991). In a cross with cultivar NCHS-1, a single QTL for PV, qPV11, was detected on chromosome 11, accounting for a considerable proportion (36%) of phenotypic variation in the population. In homozygous state, the allele from Nagcarlang increased pollen viability under CHM with around 20% (Tables 2 and 3). The continuous distribution of phenotypic values in the F_2_ population and the portion of unexplained variance indicates that undetected small-effect genetic factors are likely to be involved. The position of qPV11 was unique with respect to those identified for other traits here. Interestingly, in the F_3_ population, the NCHS-1 allele behaved dominant, suggesting that the qPV11 affects the sporophytic, rather than the gametophytic tissues. This agrees with the widely shared view that high temperature, as well as other stress factors, impair pollen development indirectly by affecting tapetal function (Parish et al. 2012; De Storme and Geelen 2014; Müller and Rieu 2016). Previouis QTLs for fruit setting under high temperature were not positioned on the tomato genome, and the subtraits affected were not studied (Grilli et al. 2007; Lin et al. 2010). The only two QTLs for pollen fertility under long-term mild heat described so far come from a study in rice. In combination, these two QTLs explained a portion of phenotypic variance comparable to that of tomato qPV11 described here (Xiao et al. 2011). Dominance characteristics were not reported for the rice QTLs. Taken together, the results indicate that pollen thermotolerance in tomato and possibly other species may be determined to a large extend by relatively few major genes.

A negative effect of heat on PN was reported before (Firon et al. 2006; Levy et al. 1978; Pressman et al. 2002; Sato et al. 2000; Sato and Peet 2005). Cultivar NCHS-1 was found to be relatively heat tolerant regarding this trait. Our QTL mapping identified one QTL for PN on chromosome 7, the positive effect coming from the NCHS-1 allele, which was also associated with AL, in line with the strong positive correlation between the two traits. The same association was recently found in wheat, where introduction of a chromosome from rye enhanced both anther size and pollen production (Nguyen et al. 2015). It seems likely that a larger anther contains more pollen mother cells and is able to support more developing pollen. Interestingly, there was no evidence for a trade-off between PV and PN, indicating that the total number of viable pollen can be increased by genetic means. Whether the number of pollen produced and the number of pollen released from the anther at anthesis are independent traits, and which of the two most strongly limits reproduction under heat, is still unclear (Sato et al. 2000; Firon et al. 2006). Future analysis of the effect of qPN7 on fruit setting ability under high temperature should shed light on this.

### Style and anther length and style protrusion

An inserted stigma is an important trait to ensure self-pollination in cultivated tomato (Rick and Dempsey 1969; Chen and Tanksley 2004). Growth at high temperature may lead to protrusion of the style out of the anther cone (Levy et al. 1978; Sato et al. 2006). SP depends on the interaction between AL and SL (Chen and Tanksley 2004). In our mapping population, SP was positively correlated with SL and negatively with AL, indicating both anther and style have effects on the extent of SP. However, the much stronger correlation between SP and SL indicates that an elongated style is the main reason for SP under heat stress. Association among these floral structure traits is also reflected by the underlying genetic architecture: both QTLs for SP colocalised with QTLs for SL. By contrast, a QTL on chromosome 2 affected both style and anther length, in the same direction, explaining why it did not contribute to variation in SP. The relatively simple genetic architecture of SP at high temperature is in line with high heritability found in previous genetic studies (Levy et al. 1978; El Ahmadi and Stevens 1979). An protruded stigma phenotype is observed often in self-incompatible wild tomato relatives to facilitate outcrossing (Chen and Tanksley 2004). To reveal how an inserted stigma evolved from the wild ancestors, Chen and Tanksley (2004) identified several QTLs for anther and style morphology. One of them (*se2.1*) was further mapped to a short region on chromosome 2. Within that region, loci for style length and stamen length were confirmed. S*tyle2.1* was cloned and turned out to encode a transcription factor regulating cell elongation; a mutation in the promoter region of *Style2.1* was responsible for low activity of the gene during flower development (Chen et al. 2007). Our QTLs on chromosome 2 affecting SL and AL under heat were mapped to the same region as *se2.1*, suggesting that the mechanisms responsible for anther and style development under normal and high temperature conditions are at least partially conserved. The two other QTLs related to SP and SL on chromosome 1 and 3 did not colocalise with any previously identified QTL under normal temperature, indicating additional, distinct mechanisms for style development upon heat stress that lead to SP.

### Formation of inflorescences and flowers

The total number of flowers produced by a tomato plant correlates with yield per plant under CMH condition (Bhattarai et al. 2016). We examined flower production by assessing the number of inflorescences and number of flowers per inflorescence. The positive correlation between these two traits suggests a common physiological basis and is in line with colocalisation of the main QTLs, qIN1 and qFPI1, on chromosome 1. A number of studies have been done on reproductive traits under normal conditions and several QTLs responsible for flowering time and FPI were published (Grandillo and Tanksley 1996; Georgiady et al. 2002; Doganlar et al. 2002). Among these, Grandillo and Tanksley (1996) reported that one of the QTLs for days to first flower was close to RFLP marker TG125 on chromosome 1. Later, a QTL for FPI was also found to be close to TG125 (Doganlar et al. 2002). This marker is very close to the QTLs identified here, suggesting that inflorescence and flower production under normal and heat condition are controlled by the same genes. Indeed, we have noticed that IN was not significantly influenced by the CMH regime (data not shown). FPI was reduced by CMH, but both parents reacted similarly (i.e. no genotype-temperature interaction), making it unlikely for heat-specific QTLs to be identified. In line with the above, El Ahmadi and Stevens (1979) found that in a germplas set, FPI under normal and mildly elevated growth temperatures correlated strongly and the traits were highly heritable.

### Implications for tomato heat tolerance breeding

The QTLs identified here are good candidates for cloning to generate knowledge on the genes involved in determining reproductive heat tolerance levels. This kind of approach is of great importance for understanding the fundamental molecular physiological mechanisms of the tolerance. However, even without this information, the identified QTLs can directly be tested in breeding programs. As an advantage, most of the QTLs mapped in this study showed additive behaviour. Nearly no epistatic interactions were found, except for the two QTLs for IN, in which case pyramiding of favourable alleles from different loci may give rise to superior offspring.

## Electronic supplementary material

Supplementary Fig. 1 Phenotype distributions for the F_2_ population. a Inflorescence number (IN); b Flowers per inflorescence (FPI); c Style length (SL); d Anther length (AL); e Style protrusion (SP); f Pollen viability (PV); g Pollen number (PN); h Female fertility (FF). Mean values of two parents were indicated by arrows. P1, Nagcarlang; P2, NCHS-1. (DOCX 91 kb).

Supplementary Fig. 2 Scatterplots of phenotypic data from mother plants and their cuttings. Correlations (r) and number of observations (n), as well as linear regression were shown for earch trait. Correlations were significant in all cases (*P* < 0.001). **a** Pollen viability (PV); **b** Pollen number (PN); **c** Style length (SL); **d** Anther length (AL); **e** Style protrusion (SP). (DOCX 138 kb).

Supplementary Fig. 3 LOD profiles of QTL mapping for all traits. For each trait, LOD scores are indicated along all 12 chromosomes. The horizontal line indicates the significance threshold. PV, pollen viability; PN, pollen number; SP, style protrusion; AL, anther length; SL, style length; FPI, flowers per inflorescence; IN, Inflorescence number. (DOCX 150 kb).

Supplementary Table 1 Primer sequences for KASP SNP genotyping assays. (DOCX 22 kb).

## References

[CR1] Akhtar S, Ansary SH, Dutta AK (2012). Crucial reproductive characters as screening indices for tomato (*Solanum lycopersicum*) under high temperature stress. J Crop Weed.

[CR2] Bac-Molenaar JA, Fradin EF, Becker FFM (2015). Genome-wide association mapping of fertility reduction upon heat stress reveals developmental stage-specific QTLs in *Arabidopsis thaliana*. Plant Cell.

[CR3] Bhattarai U, Sharma A, Das R, Talukdar P (2016). Genetic analysis of yield and yield-attributing traits for high temperature resistance in tomato. Int J Veg Sci.

[CR4] Bokszczanin KL, Fragkostefanakis S, Solanaceae Pollen Thermotolerance Initial Training Network (SPOT-ITN) Consortium (2013). Perspectives on deciphering mechanisms underlying plant heat stress response and thermotolerance. Front Plant Sci.

[CR5] Broman KW, Wu H, Sen S, Churchill GA (2003). R/qtl: QTL mapping in experimental crosses. Bioinformatics.

[CR6] Chen KY, Tanksley SD (2004). High-resolution mapping and functional analysis of se2.1: a major stigma exsertion quantitative trait locus associated with the evolution from allogamy to autogamy in the genus *lycopersicon*. Genetics.

[CR7] Chen K, Cong B, Wing R (2007). Changes in regulation of a transcription factor lead to autogamy in cultivated tomatoes. Science.

[CR8] Churchill GA, Doerge RW (1994) Empirical threshold values for quantitative trait mapping. Genetics 138:963–971 10.1093/genetics/138.3.963 PMC1206241 7851788

[CR9] Dane F, Hunter AG, Chambliss OL (1991). Fruit set, pollen fertility, and combining ability of selected tomato genotypes under high-temperature field conditions. J Am Soc Hortic Sci.

[CR10] De Storme N, Geelen D (2014). The impact of environmental stress on male reproductive development in plants: biological processes and molecular mechanisms. Plant Cell Environ.

[CR11] Doganlar S, Frary A, Ku H-M, Tanksley SD (2002). Mapping quantitative trait loci in inbred backcross lines of *Lycopersicon pimpinellifolium* (LA1589). Genome.

[CR12] Driedonks N, Rieu I, Vriezen WH (2016). Breeding for plant heat tolerance at vegetative and reproductive stages. Plant Reprod.

[CR13] El Ahmadi AB, Stevens MA (1979). Genetics of high temperature fruit set in tomato. J Am Soc Hortic Sci.

[CR14] Esten Mason R, Mondal S, Beecher FW, Hays DB (2011). Genetic loci linking improved heat tolerance in wheat (*Triticum aestivum* L.) to lower leaf and spike temperatures under controlled conditions. Euphytica.

[CR15] Firon N, Shaked R, Peet MM (2006). Pollen grains of heat tolerant tomato cultivars retain higher carbohydrate concentration under heat stress conditions. Sci Hortic (Amsterdam).

[CR16] Frank E, Harrell J (2015) Hmisc: Harrell Miscellaneous

[CR17] Gardner RG (2000). “Sun Leaper”, a hybrid tomato, and its parent, NC HS-1. Hortscience.

[CR18] Georgiady MS, Whitkus RW, Lord EM (2002). Genetic analysis of traits distinguishing outcrossing and self-pollinating forms of currant tomato, *Lycopersicon pimpinellifolium* (Jusl.) Mill. Genetics.

[CR19] Giorno F, Wolters-Arts M, Mariani C, Rieu I (2013). Ensuring reproduction at high temperatures: the heat stress response during anther and pollen development. Plants.

[CR20] Gourdji SM, Sibley AM, Lobell DB (2013). Global crop exposure to critical high temperatures in the reproductive period: historical trends and future projections. Environ Res Lett.

[CR21] Grandillo S, Tanksley SD (1996). QTL analysis of horticultural traits differentiating the cultivated tomato from the closely related species *Lycopersicon pimpinellifolium*. Theor Appl Genet.

[CR22] Grilli GVG, Braz LT, Lemos EGM (2007). QTL identification for tolerance to fruit set in tomato by fAFLP markers. Crop Breed Appl Biotechnol.

[CR23] Hall AE (1992) Breeding for heat tolerance. In: Plant breeding reviews. Wiley, p 129

[CR24] Hedhly A, Hormaza JI, Herrero M (2009). Global warming and sexual plant reproduction. Trends Plant Sci.

[CR25] Jha UC, Bohra A, Singh NP (2014). Heat stress in crop plants: its nature, impacts and integrated breeding strategies to improve heat tolerance. Plant Breed.

[CR26] Kinet JM, Peet MM, Wien HC (1997). Tomato. The physiology of vegetable crops.

[CR27] Kosambi DD (1944). The estimation of map distances from recombination values. Ann Eugenics.

[CR28] Levy A, Rabinowitch HD, Kedar N (1978). Morphological and physiological characters affecting flower drop and fruit set of tomatoes at high temperatures. Euphytica.

[CR29] Lin KH, Yeh WL, Chen HM, Lo HF (2010). Quantitative trait loci influencing fruit-related characteristics of tomato grown in high-temperature conditions. Euphytica.

[CR30] Müller F, Rieu I (2016) Acclimation to high temperature during pollen development. Plant Reprod:1–12. doi:10.1007/s00497-016-0282-x 10.1007/s00497-016-0282-x PMC4909792 27067439

[CR31] Nguyen V, Fleury D, Timmins A (2015). Addition of rye chromosome 4R to wheat increases anther length and pollen grain number. Theor Appl Genet.

[CR32] Opeña RT, Chen JT, Kuo CG, Chen HM (1992) Genetic and physiological aspects of tropical adaptation in tomato. In: Adaptation of food crops to temperature and water stress: proceedings of an international symposium. Pp 13–18

[CR33] Pachauri RK, Allen MR, Barros VR, et al. (2014) Climate Change 2014: Synthesis Report. Contribution of Working Groups I, II and III to the Fifth Assessment Report of the Intergovernmental Panel on Climate Change

[CR34] Parish RW, Phan HA, Iacuone S, Li SF (2012). Tapetal development and abiotic stress: a centre of vulnerability. Funct Plant Biol.

[CR35] Patel PN, Hall AE (1990). Genotypic variation and classification of cowpea for reproductive responses to high temperature under long photoperiods. Crop Sci.

[CR36] Pressman E, Peet MM, Pharr DM (2002). The effect of heat stress on tomato pollen characteristics is associated with changes in carbohydrate concentration in the developing anthers. Ann Bot.

[CR37] R Core Team (2015) R: a language and environment for statistical computing

[CR38] Redona E, Manigbas N, Laza M (2009). Identifying heat tolerant rice genotypes under different environments. SABRAO Journal of Breeding and Genetics.

[CR39] Rick CM, Dempsey WH (1969). Position of the stigma in relation to fruit setting of the tomato. Bot Gaz.

[CR40] Sato S, Peet MM (2005). Effects of moderately elevated temperature stress on the timing of pollen release and its germination in tomato (*Lycopersicon esculentum* Mill.). J Hortic Sci Biotechnol.

[CR41] Sato S, Peet MM, Thomas JF (2000). Physiological factors limit fruit set of tomato (*Lycopersicon esculentum* Mill.) under chronic, mild heat stress. Plant. Cell Environ.

[CR42] Sato S, Peet MM, Gardner RG (2004). Altered flower retention and developmental patterns in nine tomato cultivars under elevated temperature. Sci Hortic (Amsterdam).

[CR43] Sato S, Kamiyama M, Iwata T (2006). Moderate increase of mean daily temperature adversely affects fruit set of *Lycopersicon esculentum* by disrupting specific physiological processes in male reproductive development. Ann Bot.

[CR44] Scott JW, Olson SM, Howe TK (1995). “Equinox” heat-tolerant hybrid tomato. Hortscience.

[CR45] Shpiler L, Blum A (1986). Differential reaction of wheat cultivars to hot environments. Euphytica.

[CR46] Sim SC, Durstewitz G, Plieske J (2012). Development of a large snp genotyping array and generation of high-density genetic maps in tomato. PLoS One.

[CR47] Xiao Y, Pan Y, Luo L (2011). Quantitative trait loci associated with pollen fertility under high temperature stress at flowering stage in rice (*Oryza sativa*). Rice Sci.

[CR48] Ye C, Argayoso MA, Redoña ED (2012). Mapping QTL for heat tolerance at flowering stage in rice using SNP markers. Plant Breed.

