# Supplementary material for: Mapping quantitative trait loci for heat tolerance of reproductive traits in tomato (Solanum lycopersicum)
Source: Mol Breed. 2017 Apr 18;37(5):58. doi: 10.1007/s11032-017-0664-2 (PMC5395597; doi:10.1007/s11032-017-0664-2)
Supplement: Supplementary file 1 — Phenotype distributions for the F2 population. a Inflorescence number (IN); b Flowers per inflorescence (FPI); c Style length (SL); d Anther length (AL); e Style protrusion (SP); f Pollen viability (PV); g Pollen number (PN); h Female fertility (FF). Mean values of two parents were indicated by arrows. P1, Nagcarlang; P2, NCHS-1. (DOCX 91 kb). [file 11032_2017_664_MOESM1_ESM.docx]

**Supplementary material**

**
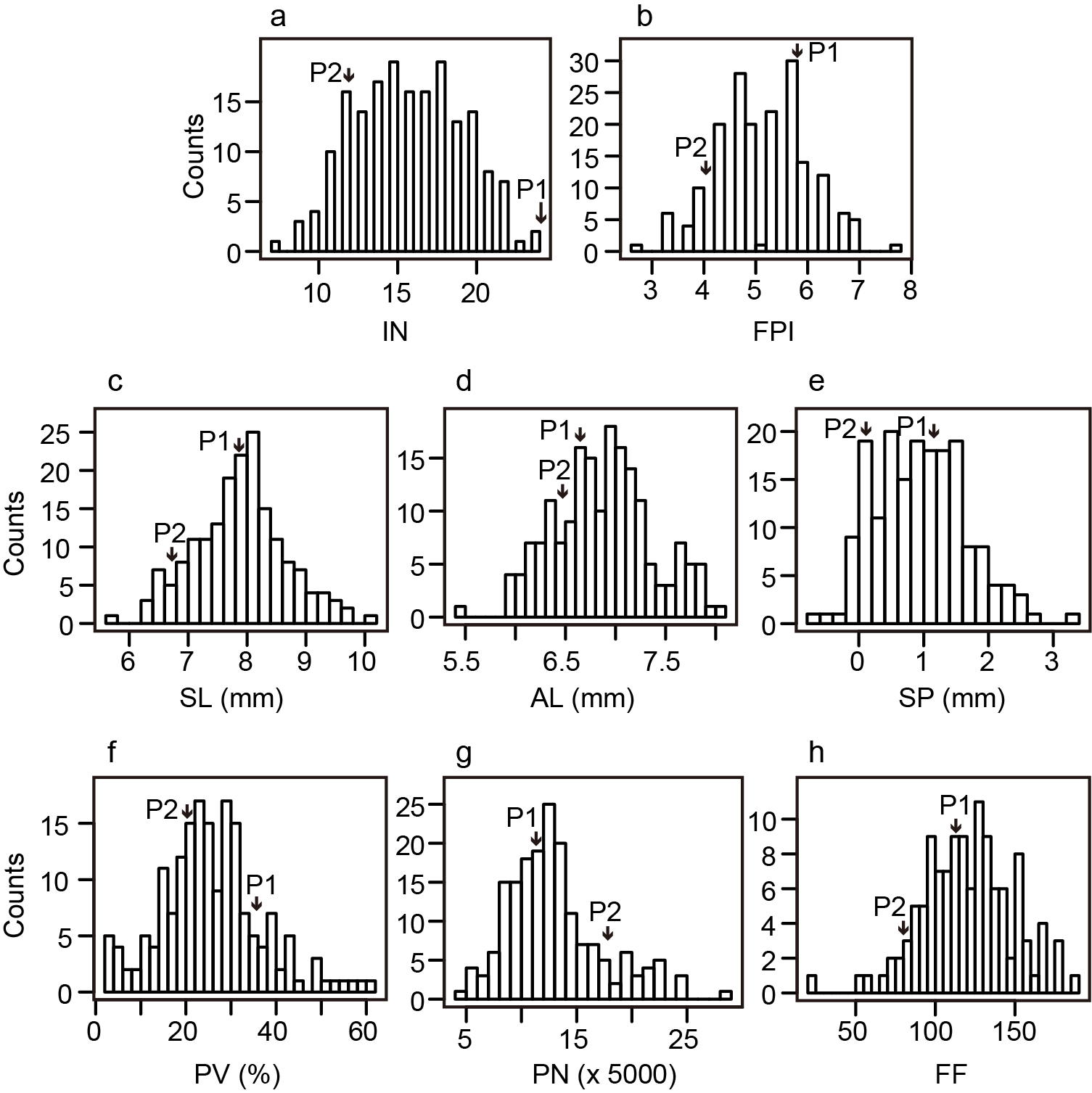
**

Supplementary Fig. 1 Phenotype distributions for the F_2_ population. a Inflorescence number (IN); b Flowers per inflorescence (FPI); c Style length (SL); d Anther length (AL); e Style protrusion (SP); f Pollen viability (PV); g Pollen number (PN); h Female fertility (FF). Mean values of two parents were indicated by arrows. P1, Nagcarlang; P2, NCHS-1.
