# Supplementary material for: Mapping quantitative trait loci for heat tolerance of reproductive traits in tomato (Solanum lycopersicum)
Source: Mol Breed. 2017 Apr 18;37(5):58. doi: 10.1007/s11032-017-0664-2 (PMC5395597; doi:10.1007/s11032-017-0664-2)
Supplement: Supplementary file 2 — Scatterplots of phenotypic data from mother plants and their cuttings. Correlations (r) and number of observations (n), as well as linear regression were shown for earch trait. Correlations were significant in all cases (P < 0.001). a Pollen viability (PV); b Pollen number (PN); c Style length (SL); d Anther length (AL); e Style protrusion (SP). (DOCX 138 kb). [file 11032_2017_664_MOESM2_ESM.docx]

**
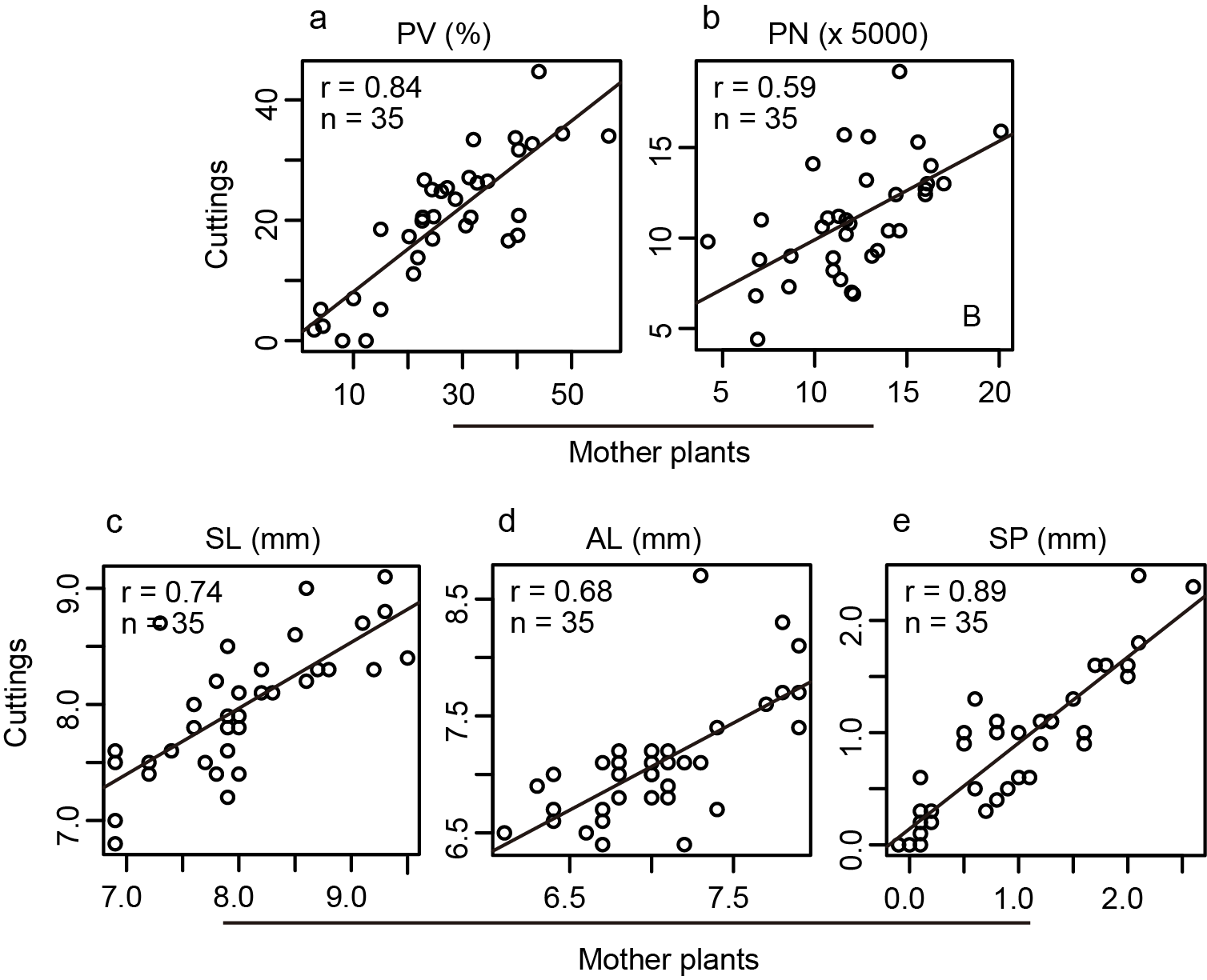
**

Supplementary Fig. 2 Scatterplots of phenotypic data from mother plants and their cuttings. Correlations (r) and number of observations (n), as well as linear regression were shown for earch trait. Correlations were significant in all cases (P<0.001). a Pollen viability (PV); b Pollen number (PN); c Style length (SL); d Anther length (AL); e Style protrusion (SP).
