# Supplementary material for: Mapping quantitative trait loci for heat tolerance of reproductive traits in tomato (Solanum lycopersicum)
Source: Mol Breed. 2017 Apr 18;37(5):58. doi: 10.1007/s11032-017-0664-2 (PMC5395597; doi:10.1007/s11032-017-0664-2)
Supplement: Supplementary file 3 — LOD profiles of QTL mapping for all traits. For each trait, LOD scores are indicated along all 12 chromosomes. The horizontal line indicates the significance threshold. PV, pollen viability; PN, pollen number; SP, style protrusion; AL, anther length; SL, style length; FPI, flowers per inflorescence; IN, Inflorescence number. (DOCX 150 kb). [file 11032_2017_664_MOESM3_ESM.docx]

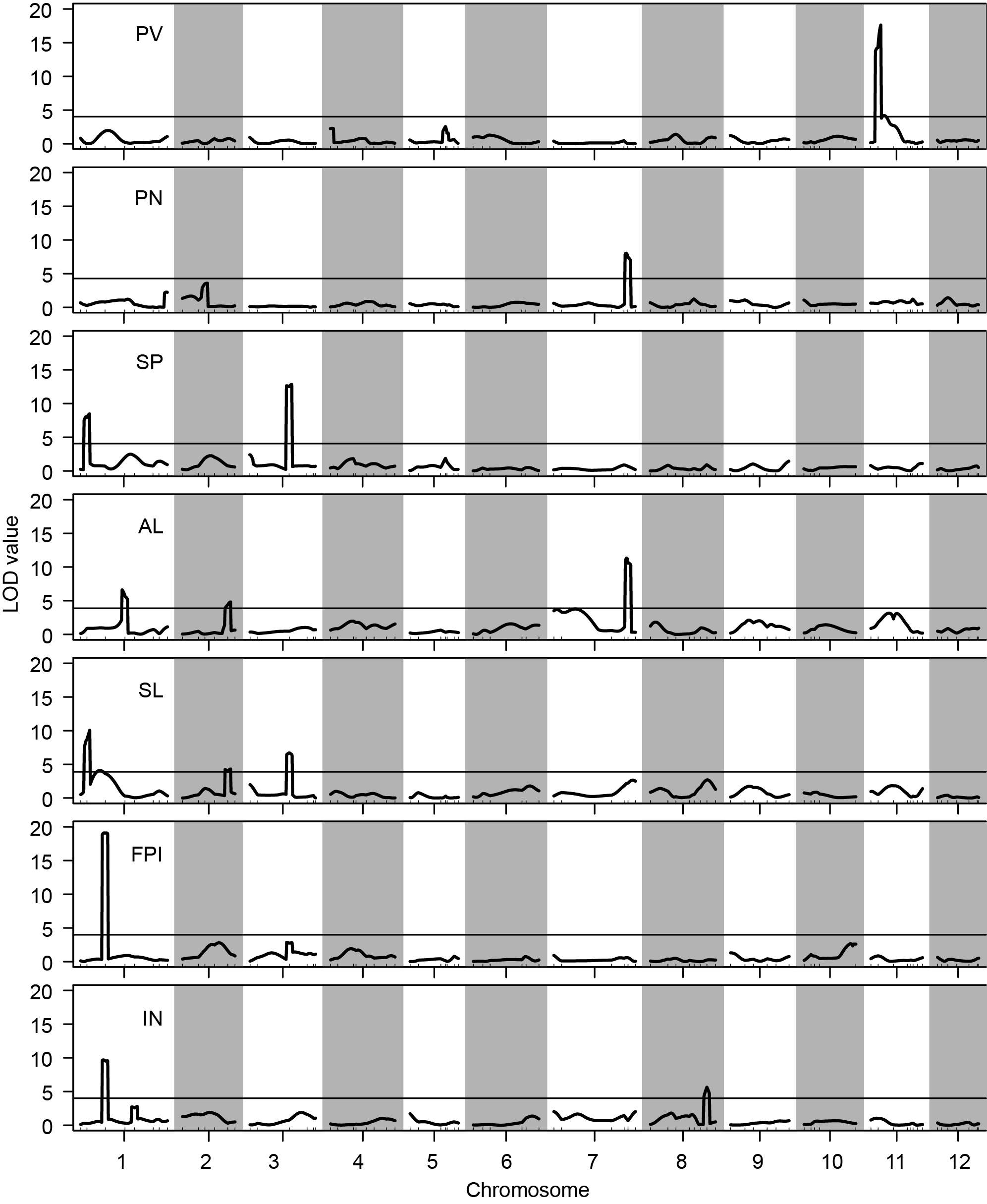


Supplementary Fig. 3 LOD profiles of QTL mapping for all traits. For each trait, LOD scores are indicated along all 12 chromosomes. The horizontal line indicates the significance threshold. PV, pollen viability; PN, pollen number; SP, style protrusion; AL, anther length; SL, style length; FPI, flowers per inflorescence; IN, Inflorescence number.
