# Supplementary material for: Mapping quantitative trait loci for heat tolerance of reproductive traits in tomato (Solanum lycopersicum)
Source: Mol Breed. 2017 Apr 18;37(5):58. doi: 10.1007/s11032-017-0664-2 (PMC5395597; doi:10.1007/s11032-017-0664-2)
Supplement: Supplementary file 4 — Primer sequences for KASP SNP genotyping assays. (DOCX 22 kb). [file 11032_2017_664_MOESM4_ESM.docx]

**Supplementary Table 1** Primer sequences for KASP SNP genotyping assays.

| **SNP Marker** | **Chromosome** | **Primer_AlleleFAM** | **Primer_AlleleHEX** | **Primer_Common** |
| --- | --- | --- | --- | --- |
| solcap_snp_sl_15055 | SL2.40ch01 | GAAAATAAAGATGAAGAAGTTAAGAGTGGAA | AAAATAAAGATGAAGAAGTTAAGAGTGGAG | GCTTTCCTGAAACATACACACTAACACAT |
| solcap_snp_sl_8704 | SL2.40ch01 | AGAACCAGGCGTTGCATTTGTAC | AGAGAACCAGGCGTTGCATTTGTAT | TCTACCACAGCGGAGCCAAAACTTA |
| solcap_snp_sl_13762 | SL2.40ch01 | TCTGTGACAACATTCTCAAAAAAGGA | CTGTGACAACATTCTCAAAAAAGGC | CAACGTTTCTTCAATAGCTTCATCACTCAA |
| solcap_snp_sl_42213 | SL2.40ch01 | CACGCGCCGCCGATTCAA | CACGCGCCGCCGATTCAG | GACACACCGACAGTTTTGTTTGTTTGTTA |
| solcap_snp_sl_15339 | SL2.40ch01 | GGAAGATATAGAAGGCATGGAGG | GGGAAGATATAGAAGGCATGGAGA | GTAGCACTCTTGGGGAGTATTTCCTT |
| solcap_snp_sl_40366 | SL2.40ch01 | GTAAACTCTGCTGGTGCTTCAAC | CTGTAAACTCTGCTGGTGCTTCAAT | GAGTGAGAAGAAATTGCTTTAAAAGCAGAT |
| solcap_snp_sl_19481 | SL2.40ch01 | AAGGCCCGAGGCAAAAACAGCA | GGCCCGAGGCAAAAACAGCG | TCTCTATGAGCGCGAGCAAGTTCTT |
| solcap_snp_sl_48121 | SL2.40ch01 | AGCAAATAATGTCAATGTGCATATCGA | AGCAAATAATGTCAATGTGCATATCGC | TATGATGAGAGGAGTGCTCCGGAAA |
| solcap_snp_sl_28338 | SL2.40ch02 | CATTCCTTTGGGATCTTTTGTGGCA | CCTTTGGGATCTTTTGTGGCG | ATACGTGGCCAACCCTTTCAGTGAT |
| solcap_snp_sl_8402 | SL2.40ch02 | ATGTTGGTCAAGAACAGTTTGGCCA | GTTGGTCAAGAACAGTTTGGCCG | TGAAGCCTGTATGGTGTCATATTTGCAAA |
| solcap_snp_sl_8505 | SL2.40ch02 | TACAAATTCGTGCAAGCGGGG | ATCTTACAAATTCGTGCAAGCGGGA | CCCTGTGTAGTATCATTTTGAAAGATACAA |
| solcap_snp_sl_13516 | SL2.40ch02 | TTCCTTAGTAGCACTGCTCCATGT | CCTTAGTAGCACTGCTCCATGG | GAGAACAATGGCCAAGCTCAAGCTA |
| solcap_snp_sl_18447 | SL2.40ch02 | CGCTTCTTTAGATTGAAACAGAGTGT | CGCTTCTTTAGATTGAAACAGAGTGC | CTTCATATGCCTGCTTTTGGAGTGAAATA |
| solcap_snp_sl_17161 | SL2.40ch02 | CCAGAAATCTTTTACAAGCAATAAATGTTCT | CCAGAAATCTTTTACAAGCAATAAATGTTCA | GCAAAAGTAGATCCAGAATCCAGGTTAAT |
| solcap_snp_sl_36287 | SL2.40ch02 | ATCGCATTATCTTCTGTCGTTTCTAC | GTATCGCATTATCTTCTGTCGTTTCTAT | CCAAGTATGGTTGTGTTAGGCGGTT |
| solcap_snp_sl_32389 | SL2.40ch02 | CGGTGGTTCAAAGACCTCACG | GTCGGTGGTTCAAAGACCTCACA | AATACGAGCTTGCAGGCTGCTGAAA |
| solcap_snp_sl_9666 | SL2.40ch03 | GTCAAAGCTTTTAGCTAAAATGTCTCGA | CAAAGCTTTTAGCTAAAATGTCTCGG | CCTTAAGTGTTTTGATGGGCACGTTTTAT |
| solcap_snp_sl_14354 | SL2.40ch03 | GGTGCCATTCAAGTTAATTGCTTGATT | GTGCCATTCAAGTTAATTGCTTGATC | GGGTTTCGGTTCAGAAAAACATCAATGAA |
| solcap_snp_sl_18590 | SL2.40ch03 | CAGTCTAACATTATTTTACAAAGAGAGAGAA | AGTCTAACATTATTTTACAAAGAGAGAGAG | TCAACACCAACACTTCCACTTTACTGATT |
| solcap_snp_sl_7942 | SL2.40ch03 | GGTTAAGTGCCAAGAACATGGAC | CTGGTTAAGTGCCAAGAACATGGAT | CCCGAAAGAGTTAATTAAACATGCAATGAA |
| solcap_snp_sl_62348 | SL2.40ch03 | TGCTCATAAAGATGAAATAAACAGAAGAC | CTTTGCTCATAAAGATGAAATAAACAGAAGAA | GTTTGAAGGGACAAAATGTGGACTACATT |
| solcap_snp_sl_34106 | SL2.40ch03 | TCTCAGGTGAACTTCTTCATGCT | CTCAGGTGAACTTCTTCATGCC | CCAGTGAGTGAAGAAGGAATAGTAGATTT |
| solcap_snp_sl_33830 | SL2.40ch03 | CACAAAACATGGCAAAGTTAAGTACAATTA | CACAAAACATGGCAAAGTTAAGTACAATTT | GAGAAAAAGAACCTGCATTTTGCGGTT |
| solcap_snp_sl_36532 | SL2.40ch03 | GAAGACGGACAGAATCCTCG | ATCTGAAGACGGACAGAATCCTCA | GGAAGACTCTATGCTTTGAAAGTGATCTA |
| solcap_snp_sl_63869 | SL2.40ch04 | GGTGCTGATATATTAGCTGATAGACTA | GTGCTGATATATTAGCTGATAGACTC | AAGAGGTACCTTTTCGGCAATAGAGTTTT |
| solcap_snp_sl_21335 | SL2.40ch04 | CACCTGATAACTCACTTTATGGTCCA | ACCTGATAACTCACTTTATGGTCCG | TGTGAACATTGAGACAGTTGAGATGGAAT |
| solcap_snp_sl_16992 | SL2.40ch04 | CAAATCAGTTGAGCAATTAAACTCACACT | AATCAGTTGAGCAATTAAACTCACACG | CAGTTGTACGAGGGCTCGTGGTA |
| solcap_snp_sl_17641 | SL2.40ch04 | GTGATATATGAAGAAGAGCTTGGCG | GTGATATATGAAGAAGAGCTTGGCT | GGTTCATCTGTGACTTCCACCACTA |
| solcap_snp_sl_3106 | SL2.40ch04 | ACGGAATCGATTGCTCCGACGA | CGGAATCGATTGCTCCGACGG | CTGGCGAAGAAGACTGCATCGTTAA |
| solcap_snp_sl_11543 | SL2.40ch04 | GAACGATTGATTAAACCTTTAGACTCAC | GAACGATTGATTAAACCTTTAGACTCAT | TTAGCAACAGCTCAAATCACCTCATGAAA |
| solcap_snp_sl_3748 | SL2.40ch04 | AGCAGCACTACTCAAAGCTA | GCTAGCAGCACTACTCAAAGCTG | ATAGCTGCTGGACTCCATTCTGGAT |
| solcap_snp_sl_4024 | SL2.40ch04 | CAACACTTAAATTTTGACATGTCCTAGAAA | CAACACTTAAATTTTGACATGTCCTAGAAT | GAGCAAAGATAGAGAAAGTTCTTGATCCTA |
| solcap_snp_sl_19103 | SL2.40ch05 | GCCATTGGCCGGGATAACTCTA | CCATTGGCCGGGATAACTCTG | CTTGAGCACCAAGCGAGAGAACTTA |
| solcap_snp_sl_13482 | SL2.40ch05 | TTGCCTATCAAAGCTCCGGCG | CTTTGCCTATCAAAGCTCCGGCA | GGGCTTACCTCTAGCTATAGTGGTT |
| solcap_snp_sl_16137 | SL2.40ch05 | ATTTCCCACAAAAGGGTCTGTCC | GATTTCCCACAAAAGGGTCTGTCA | TTAAGACATGATCAGGTGCGTGTTCTTA |
| solcap_snp_sl_12207 | SL2.40ch05 | CACCGACTCTGCTCTCTGAGA | CACCGACTCTGCTCTCTGAGG | TGCTACTGCCAATGGAGCCACTAAA |
| solcap_snp_sl_123 | SL2.40ch05 | TAGTTCAACATCCTCCAACGAACG | AGTTCAACATCCTCCAACGAACA | TTGTGGTCAGATCAGAGCCCTCTAT |
| solcap_snp_sl_249 | SL2.40ch05 | AATCCAGTCTCCCAAACATCTTCAATA | CCAGTCTCCCAAACATCTTCAATG | CTCAGTAGTTTGATTCAGCGAGAAATCTA |
| solcap_snp_sl_369 | SL2.40ch05 | CTTGCAAGCAAATGGAAGACTTC | CCTCTTGCAAGCAAATGGAAGACTTT | CTTGCGATAATGAGAACGTGATTGAACTT |
| solcap_snp_sl_35256 | SL2.40ch06 | GAAAGACTAGCAGAGTGCAGCAG | GAAAGACTAGCAGAGTGCAGCAA | CTTCTTGCTAGTTGTAGGGAAGATCTATA |
| solcap_snp_sl_101043 | SL2.40ch06 | CATTCTGTTTTATTTGTTCCTTACTTGTCTA | CATTCTGTTTTATTTGTTCCTTACTTGTCTT | CTTGTCTTGCACCTGTAATTTACATTCAAA |
| solcap_snp_sl_2622 | SL2.40ch06 | CTTGTTAAGGTCTAATTTTGCTCGGT | CTTGTTAAGGTCTAATTTTGCTCGGG | CCCCAGGGCATGTACTAATGGAATT |
| solcap_snp_sl_1337 | SL2.40ch06 | GCAGAGTTCAAAGGGGGGG | CTGCAGAGTTCAAAGGGGGGA | TCAAAATCCCCTCCTCCCTCTCTTT |
| solcap_snp_sl_19915 | SL2.40ch06 | CAGACAAATTTAACCTCAACTTCCCA | CAGACAAATTTAACCTCAACTTCCCG | AAGGACTTCTCTGGCTTGGTGCATA |
| solcap_snp_sl_19898 | SL2.40ch06 | GCAGGATTATTGCCGGTGGTCT | CAGGATTATTGCCGGTGGTCC | GTTTCTTTGTGCTTGCATTTATGGGCTAT |
| solcap_snp_sl_31730 | SL2.40ch06 | ACATCGTTGAAATCCAACTGTCCT | ACATCGTTGAAATCCAACTGTCCC | GATGAAAGGGTATCATCTCCACCAGTA |
| solcap_snp_sl_31712 | SL2.40ch06 | GCAATGTTAAATACCTGTTATCAGCCA | CAATGTTAAATACCTGTTATCAGCCG | CAGGTTAAATACGTAGTAGTTATTCAGGAT |
| solcap_snp_sl_11221 | SL2.40ch07 | AGCCTCCGGGAAAGTTGAGTCA | AGCCTCCGGGAAAGTTGAGTCT | GGCTCTACTTTAGCTTCATCAGTTTTCTT |
| solcap_snp_sl_11171 | SL2.40ch07 | GGAAGAGTTCCTCGTACACCG | GGGAAGAGTTCCTCGTACACCA | GGCTATCCAAAGTGAAATGTCAAACACTT |
| solcap_snp_sl_70080 | SL2.40ch07 | GTGATGCCGTCGATGAGGGC | GGTGATGCCGTCGATGAGGGT | ACGTGCTTCGTTTTCTTCAATACACTAAAA |
| solcap_snp_sl_53585 | SL2.40ch07 | AGTTTTAATTCTTACGCACTCTATGCTTAT | GTTTTAATTCTTACGCACTCTATGCTTAC | CTCGGGCAAGTAGCCAAATGGTAT |
| solcap_snp_sl_55513 | SL2.40ch07 | TCTTTCTCAGGCTGGTGTGATA | CTTTCTCAGGCTGGTGTGATG | GCTCAGCTGAATGGATGGTGTCATT |
| solcap_snp_sl_12139 | SL2.40ch07 | GGCCACTCAACATCGCCGGAT | GGCCACTCAACATCGCCGGAA | CTGACCTCGAAAATTCCGACCCAAT |
| solcap_snp_sl_37060 | SL2.40ch07 | AATCTGCATCACTATGCTGTCGATTAA | CTGCATCACTATGCTGTCGATTAC | GAATCAGATCATGTGGATTCTCCTTTGAA |
| solcap_snp_sl_37030 | SL2.40ch07 | AAATTGCATGGTTTCAGCTCTGTGC | AAAAATTGCATGGTTTCAGCTCTGTGT | CTTTCCTGGACAACTCAGACGTAGTA |
| solcap_snp_sl_24384 | SL2.40ch08 | AATCATCAATCCACATTTATGCAGCTC | AATCATCAATCCACATTTATGCAGCTG | AGTCCCAGTAGGGTCGTCAATGAA |
| solcap_snp_sl_13899 | SL2.40ch08 | AAATATTATGTAGTGATCTTTGATGCAAATGTA | ATTATGTAGTGATCTTTGATGCAAATGTG | CCAGAGCTTCCATTGCTTCTCATATTTAA |
| solcap_snp_sl_13456 | SL2.40ch08 | CATCGGAGGAGATTCAAGAGGT | CATCGGAGGAGATTCAAGAGGC | GGTCGGGTCGGGTTTGAATCTTTTT |
| solcap_snp_sl_64502 | SL2.40ch08 | CGTGTTTTTGATCCGAATGTCCAC | CCGTGTTTTTGATCCGAATGTCCAT | ATCTCAAAGAATAACTTCTATCAATGATTT |
| solcap_snp_sl_21430 | SL2.40ch08 | ATAAGTTGTACTTACATTTTTTCGCCATCA | AGTTGTACTTACATTTTTTCGCCATCG | CACCTGAAACAACACCATTATCTCTGTAA |
| solcap_snp_sl_34763 | SL2.40ch08 | TCCAAGGTCACTGGTATAATGCG | CTTCCAAGGTCACTGGTATAATGCA | CCTAATGTGCTTCACCAGAAAGGCAT |
| solcap_snp_sl_15446 | SL2.40ch08 | AAGTAAAATCCCTAGCAACACTACCT | AAGTAAAATCCCTAGCAACACTACCA | GAGGGAAGGAAATCAAGATTTTTGAGACAT |
| solcap_snp_sl_36988 | SL2.40ch08 | AGCTTCATTAGATTACAGTTGTATTTCCTT | AGCTTCATTAGATTACAGTTGTATTTCCTA | GCCGGAGCTACACCGGAGAAT |
| solcap_snp_sl_28415 | SL2.40ch09 | TCAAGTTATGCTCGAGCTTTGTCAAA | CAAGTTATGCTCGAGCTTTGTCAAG | TTCAGGCTCAGTGAAGTGATAATCATGTT |
| solcap_snp_sl_14676 | SL2.40ch09 | AAGGTATTCCGACCCTATCTTGCTT | GGTATTCCGACCCTATCTTGCTC | GTCGCACCTGTGCTGAATACTTCTA |
| solcap_snp_sl_39804 | SL2.40ch09 | CAACGACTGCAGGTAACTTTCCG | CAACGACTGCAGGTAACTTTCCA | CTGGTGACGTTTTGGTTGATTCTATGAAA |
| solcap_snp_sl_16585 | SL2.40ch09 | AAACAAGAACTTGAAAGCCCACCT | AAACAAGAACTTGAAAGCCCACCC | GGTGCAGAAAGAGATTCGTCAGCAA |
| solcap_snp_sl_3355 | SL2.40ch09 | CTTCCTCTTCTTCATGTCCTCTCT | CCTCTTCTTCATGTCCTCTCG | GATCTATGATTTTGCGAAGTGAAGTGAAAT |
| solcap_snp_sl_7829 | SL2.40ch09 | CACGTTCGTGCAATCGTTGCTCA | ACGTTCGTGCAATCGTTGCTCG | ATGATCTGTTCTGGAGTTGTTGCTCAAT |
| solcap_snp_sl_36852 | SL2.40ch09 | CAGACAACAAGGGTGTGGCTCT | AGACAACAAGGGTGTGGCTCC | TGAACTGGAAGAATTTTAGAGGGATGATAA |
| solcap_snp_sl_25745 | SL2.40ch09 | CCTAGCCGGGCGTTGAAATTGT | CTAGCCGGGCGTTGAAATTGG | GAGATGTTAAGACAAGCAATGTGATGCTT |
| solcap_snp_sl_46059 | SL2.40ch10 | GATGCTCATCCAATGCATCAAACTCT | ATGCTCATCCAATGCATCAAACTCG | CCCTATGTATCATCATCTACGTAACTCAA |
| solcap_snp_sl_17859 | SL2.40ch10 | CATATTCATACTCACTCCGCTTCTCT | ATATTCATACTCACTCCGCTTCTCC | CACAGAGAGCTTAGTTTGATGAAAGCTTT |
| solcap_snp_sl_30517 | SL2.40ch10 | AGCCGCAGAGGTAGTAGTTCC | CAGCCGCAGAGGTAGTAGTTCT | TCCTCGCTGACACAGTCTCCGA |
| solcap_snp_sl_25580 | SL2.40ch10 | AATACCATTTTTTTCAGCAAATCCAAGAGA | ACCATTTTTTTCAGCAAATCCAAGAGC | GCAAGTTGTTACGCGATTAGTAAGACATT |
| solcap_snp_sl_18726 | SL2.40ch10 | GACAGCAAACCTAGATGAGAAATTACA | GACAGCAAACCTAGATGAGAAATTACT | CTCGTGAGGAAGAAGATGGGCTTTT |
| solcap_snp_sl_61192 | SL2.40ch10 | ATCATAGAAATGACTTCCGCGCGT | CATAGAAATGACTTCCGCGCGC | TCCCACTATAAGAAATTGAATTCCAGTGAA |
| solcap_snp_sl_8858 | SL2.40ch10 | TTCATTCACCCGTACCCTTTCCA | CATTCACCCGTACCCTTTCCC | CGGAGTTGATTCGGAGAGACGAAA |
| solcap_snp_sl_8808 | SL2.40ch10 | GTACAGATAACAAACCACAATCAGTGA | ACAGATAACAAACCACAATCAGTGG | CTTCAGGTGATTGAACCTGCACTCTA |
| solcap_snp_sl_36173 | SL2.40ch11 | TTGTTAGGGTATTTTTGTATTGTTTGCATTTA | GTTAGGGTATTTTTGTATTGTTTGCATTTG | TCCAAAAGGCGGGACAAGGAAGTT |
| solcap_snp_sl_36066 | SL2.40ch11 | TACTCGGCTCAGCTGGAATT | CTTACTCGGCTCAGCTGGAATC | GGAGTTGTGCCGGCTATTAAGGATA |
| solcap_snp_sl_9486 | SL2.40ch11 | AGCCACCAGATGATTCATCCTCTAA | CCACCAGATGATTCATCCTCTAG | CTGCTCAAGCTTTATAACCCATTTCTCTA |
| solcap_snp_sl_6125 | SL2.40ch11 | AACTCTGTATGATGTCTCGGTAGAAT | ACTCTGTATGATGTCTCGGTAGAAC | AGTATAACTTGAGGCAGCTTACCTCTAAA |
| solcap_snp_sl_19149 | SL2.40ch11 | GAGTCTGAATTCGTGCTATATTTGATC | TGAGTCTGAATTCGTGCTATATTTGATA | GAACATCATTTTTCTGAGATGGTCTGTAAT |
| solcap_snp_sl_5970 | SL2.40ch11 | TTCTACTGAACTACTCCTATTATCTAAT | TCTACTGAACTACTCCTATTATCTAAG | CTGCAAGGAATAAGAATTGCAATGCTGTT |
| solcap_snp_sl_100996 | SL2.40ch11 | CATGATGATCGTCAGTTTCACAAG | CTCATGATGATCGTCAGTTTCACAAA | CAGGTGTGATTTCACCTCTGGAATAATTT |
| solcap_snp_sl_2711 | SL2.40ch11 | GAAGACAAGCGAGCGTTGGCT | GAAGACAAGCGAGCGTTGGCC | GCTTAGCCTTCTCCTCATAGTTAAACAT |
| solcap_snp_sl_17703 | SL2.40ch12 | CACTTCGTGGTGAAGAGCTTATTAGA | ACTTCGTGGTGAAGAGCTTATTAGG | ACTTGTTTCCTCTCTTTAACCATCCGTTT |
| solcap_snp_sl_3165 | SL2.40ch12 | GATCAGTGTGTAGAATTTAAATGCTATAACT | ATCAGTGTGTAGAATTTAAATGCTATAACG | CAAAAGCAAGCTTTCGTCTCTCTCGAT |
| solcap_snp_sl_12656 | SL2.40ch12 | ACGCCAACAAGTCTGAGTTTGGAAT | GCCAACAAGTCTGAGTTTGGAAC | CCTTTGCGAATACTGGAATTGAGCTTAT |
| solcap_snp_sl_1572 | SL2.40ch12 | GGTTCCTTGCTGCTGTGAGAAGA | GTTCCTTGCTGCTGTGAGAAGG | GAGTATAAGTTCCACAATGGAAATGCCAA |
| solcap_snp_sl_3112 | SL2.40ch12 | AACCATCTTCTTCGGTAACCCAACA | CCATCTTCTTCGGTAACCCAACG | GCACCGGCAATACTCTGAGAAGTTT |
| solcap_snp_sl_31953 | SL2.40ch12 | AAAAGAATGTTTGGTGATCTTGGAAACTAT | AGAATGTTTGGTGATCTTGGAAACTAC | GCACATAAACCACCAAGAAAGAACCAATT |
| solcap_snp_sl_14428 | SL2.40ch12 | CTTCTAACATTATGTCATTGAATGATTTTTTC | CTTCTAACATTATGTCATTGAATGATTTTTTT | GAGGAATGGTGCTAGAACTAGAACCTT |
| solcap_snp_sl_6526 | SL2.40ch12 | ACACGCCATGGGTTAGAAAATAATCAA | CACGCCATGGGTTAGAAAATAATCAG | TGGGCCTAAACCATTTTTATACGGTTGAT |
